# Situational analysis and future directions for medicine retail outlets: compliance with pharmaceutical regulatory standards in Ethiopia

**DOI:** 10.3389/fmed.2025.1452875

**Published:** 2025-03-12

**Authors:** Yesuneh Tefera Mekasha, Habtamu Getahun, Addisu Afrassa Tegegne, Gemmechu Hasen

**Affiliations:** ^1^Department of Veterinary Pharmacy, Pharmaceutical Quality Assurance and Regulatory Affairs, University of Gondar, Gondar, Ethiopia; ^2^Oromia Regional Health Bureau, Addis Ababa, Ethiopia; ^3^Department of Pharmaceutical Chemistry, School of Pharmacy, College of Medicine and Health Sciences, University of Gondar, Gondar, Ethiopia; ^4^Jimma University Laboratory of Drug Quality (JuLaDQ), Jimma, Oromia, Ethiopia; ^5^Clinical Trials Directorate, Armauer Hansen Research Institute, Addis Ababa, Ethiopia

**Keywords:** review, medicine retail outlets, poor-quality drugs, compliance, regulatory standards, EFDA, Ethiopia

## Abstract

**Background:**

Medicine regulation is essential for safeguarding the safety, efficacy, and quality of pharmaceutical products available in medicine retail outlets (MROs). It ensures that medicines, whether sourced locally or internationally, comply with stringent quality standards and regulatory protocols to protect public health. Effective regulation enhances trust in pharmaceutical markets by ensuring the availability of safe and effective medications. However, the regulatory framework within healthcare facilities, particularly in MROs where significant drug-related information is exchanged, is often underperforming. This issue is particularly pronounced in low-and middle-income countries like Ethiopia, where MROs play a critical role as the initial point of healthcare contact. This review aims to assess the level of compliance with pharmaceutical regulatory standards in medicine retail outlets (MROs) in Ethiopia and to evaluate the enforcement of these standards in the country’s pharmaceutical market.

**Methods:**

The review utilized online databases such as PubMed, and Web of Science to collect relevant studies and reports. It focused on issues of regulatory compliance in MROs in Ethiopia, identifying gaps in practice, drug quality, and adherence to national guidelines set by the Ethiopian Food and Drug Authority (EFDA).

**Results:**

The review revealed significant regulatory compliance issues within Ethiopian MROs, leading to the distribution of poor quality medicines. It was found that drugs obtained from non-compliant outlets failed to meet quality control standards outlined in drug monographs. Many MROs and pharmacy professionals were found to be non-compliant with EFDA guidelines. Additionally, dispensing and storage practices in MROs were not in line with EFDA regulations and required improvements.

**Conclusion:**

The review underlines the need for regulatory enforcement in Ethiopia health settings to address issues of non-compliance and the distribution of poor-quality drugs. It suggests that regulatory bodies should enhance inspection measures and provide opportunities for continued professional development for pharmacy staff and regulatory personnel. Collaboration among regulatory authorities, government, professionals, and academic researchers are pivotal to improving compliance and ensuring the availability of safe, effective, and quality medicines within medicine retail outlets and then protecting public health.

## Introduction

The primary objective of medicine regulation is to safeguard public health by guaranteeing that medicines available in both national and international markets are safe, effective, and of high quality and are utilized in compliance with proper protocols (1). Effective governance plays a key role in enhancing access to medicines and boosting health systems. Proper governance in the pharmaceutical field involves creating and enforcing suitable policies and protocols to guarantee the proper, efficient, and ethical oversight of medicine regulation in a transparent, accountable, and lawful manner (2–4).

Medicine outlets play a critical role as the initial healthcare touch point in low-and middle-income countries (LMICs). However, their regulation is often inadequate, and the regulatory and pharmacy personnel there may lack the necessary qualifications to offer appropriate advice. This situation poses a risk of unrestricted availability to potentially harmful products, such as unnecessary antimicrobials (5). In Ethiopia, the pharmaceutical market faces challenges with unqualified personnel and inadequate resources, hindering compliance with regulatory standards. A study by Sultan et al. in 2016 identified a notable scarcity of competent human resources for medicine regulation in Ethiopia. Low salary, unappealing career structure, and lack of incentives were primary factors contributing to the challenges of recruiting and retaining qualified pharmacy as well as regulatory personnel within the regulatory system (6).

### Regulatory compliance and medicine retail outlets

The medicine retail outlets must adhere to the regulatory system to prevent unauthorized medications from being distributed to the public, which may result in drug resistance and unnecessary expenses for patients (7). A survey in Ethiopia found that 41.0% of respondents reported the presence of unauthorized pharmaceutical businesses (6). Effective implementation of pharmaceutical regulatory systems in Africa requires a collective effort and the establishment of robust control mechanisms for medicine outlets, supported by strong regulatory authorities across multiple countries. In Kenya, many specialized drug shops (SDS) failed to meet regulatory requirements, with only 12% having a refrigerator and 22% having a separate dispensing area. Less than half had at least one staff member with a pharmacy qualification, and non-compliance was more prevalent in rural areas (8). Furthermore, less than half of the SDSs had at least one staff member with a pharmacy qualification (46%), and less than a third of the operators interviewed were aware of the name of the law that governs pharmacy operations. Non-compliance with regulations was more prevalent among specialized drug shops located in rural areas, those that did not employ staff with pharmacy qualifications, and those whose operators were unaware of the pharmacy law. Future investigations should also delve into the reasons behind the lack of regulatory compliance, despite the occurrence of regular inspections, from this perspective. Tanzania also experienced a high rate of health-related regulation violations in drug shops, with many establishments operating without proper permits and selling prescription-only medicines and unpackaged tablets illegally (9).

### Compliance perspectives on the practices and adherence of medicine retail outlets to regulatory standards

Private drug retailers, hospital pharmacies, and community pharmacies play a critical role in sharing healthcare information within the pharmaceutical sector. Community pharmacies, in particular, act as essential healthcare providers in various regions globally, catering to a significant portion of the population in underprivileged nations (10). The study conducted in Pakistan encompassed the delivery of healthcare services, primarily targeting the private sector. Around 76% of the overall healthcare expenses are financed through direct payments made by individuals. The findings from Pakistan revealed that 133 (35.8%) pharmacies were granted narcotics licenses, with 66 (17.8%) pharmacies having expired licenses, and the validity of 87 (23.0%) licenses remaining uncertain (11). A mere 113 (30.5%) pharmacies were found to be fully compliant. While 80% of the pharmacies possessed refrigerators for storing medicines, only 284 (76%) of these refrigerators were operational. Detailed medicine purchase records with warranties were maintained by 210 (56.6%) pharmacies. Furthermore, none of the pharmacies were fully adhering to the legal requirements concerning licensing, premises, storage, documentation, narcotics section, drug labeling, and prescription verification (11).

The study revealed that the studies were carried out in 10 different countries, with the majority of them being participatory research studies. The findings indicated that fire safety had the lowest aggregate percentage compliance at 0.9%. Moreover, controlled substance, climate, light, ventilation, temperature, stock, and bookkeeping operations all exhibited overall aggregate percentage compliance levels below 50% (12). Despite the presence of basic controls and measures, the review identified a lack of compliance in numerous good storage practices operations. The analysis further reinforced the notion that the presence of substandard drugs can be anticipated in cases of poor adherence. Consequently, a recent examination of existing literature revealed that the rate of antibiotic ineffectiveness (the percentage of samples in a prevalence study that did not pass at least one quality assessment) mounted at 15.7% for private pharmacies (13). Additionally, a study conducted in Ethiopia showcased the integration of sampling and testing. Within the scope of this review, the analysis of amoxicillin samples acquired from non-compliant retail establishments indicated a slight degradation when compared to compliant retail outlets. However, it is worth noting that the degradation was still within the acceptable limits in compliant retail outlets set by pharmacopeial standards (14).

### Regulatory framework in African medicine retail outlets

The lack of effective implementation of pharmaceutical legislation in Africa poses significant regulatory challenges, leading to the widespread distribution of poor-quality drugs across the continent (15). Historical data published in the past ten years shows that in sub-Saharan Africa, the availability of formal pharmacies is quite scarce, with only a few pharmaceutical retailers operating in this region. However, there are a significant number of general stores that offer a wide variety of household products and groceries. Additionally, small drug shops play a critical role in providing medicines in various parts of East and West Africa, such as Tanzania (9, 16), Uganda (17), Eritrea (18), Nigeria (19), and Cameroon (20).

The quality of pharmaceutical products could be called into question as a result of this situation. An ineffective drug regulatory system could lead to the distribution of substandard drugs throughout African pharmaceutical markets (6). While poor quality medicines are found in various regions globally, Africa carries a substantial share of this issue on a global scale. The prevalence of poor-quality medicines in the African region is significantly high, accounting for 18.7% of falsified and substandard medicines among low-and middle-income countries worldwide (21).

### Poor quality medicines in the global pharmaceutical market

The global market for these counterfeit and substandard medicines is estimated to be valued at US$65–200 billion annually (22). Additionally, the World Custom Organization has reported that approximately US$200 billion worth of fake and potentially dangerous medicines are being sold worldwide each year (23). The prevalence of poor-quality medications (8) rises due to lack of a strong regulatory system due to factors like inadequate oversight, high demand, complex supply chains, resource constraints, and low public awareness. Strengthening regulations with technology, education, and cross-border collaboration is essential to ensure medication safety and efficacy (24).

It is a proven fact that the occurrence of low-quality pharmaceuticals continues year after year, particularly in nations with resources limited region such as East Africa (25). In a systematic review conducted by Tegegne AA et al. in 2024, it was observed that 22.6% (151/669) of the samples of antimicrobials failed at least one quality test. The prevalence of failure was found to be 17% (73/432) in antibiotics, 24% (41/171) in antimalarials, and 56% (37/66) in anthelmintics (25). Therefore, it is significant for pharmaceutical health facilities worldwide, including those in African continents, to adhere to regulatory measures such as personnel requirements, premises standards, laboratory protocols, good manufacturing practices (26), good distribution practices (27), good storage practices, and good transport practices. Additionally, implementing good dispensing practices is pivotal in order to improve the quality of pharmaceutical services in the health facility (28).

### Overview of medicines regulatory systems in Ethiopia

Medicines are essential to health care and must be available to the inhabitants of every country (29). Medicines regulation aims to ensure that medicines on national markets and in international commerce are safe, effective and of good quality, are accompanied by complete and correct product information, and are manufactured, stored, distributed and used in accordance with good practices (30). Challenges that threatens the safety, efficacy and quality of medicines at every stage of their life cycle: Weaknesses in research and development, deficiencies in dosage form design, varying standards in ongoing production, damage during transport and storage, and inadequate use of products by prescribers and patients (31). An effective system must therefore provide the full range of regulatory functions, covering every stage of the cycle. The main functions of an National Medicine Regulatory Authority (NMRA) include control of pharmaceutical products by registration and post-marketing surveillance (quality monitoring and pharmacovigilance), as well as control of activities by licensing and inspection of manufacturers, importers, exporters, wholesalers, distributors, pharmacies and retail outlets, control of clinical trials and control of promotion of pharmaceuticals (32).

Ethiopia is one of the sub-Saharan African countries where the pharmaceutical sector is being guided by a national medicine policy (33). *The Pharmacists and Druggists Proclamation No 43/1942*″ was the basis for pharmaceutical regulation where both pharmacists and druggists together with the facilities where they practiced were regulated. Comprehensive regulation of the pharmaceutical sector was started in the early stages by a regulation called “*Pharmacy Regulation No. 288/ 1964*,” which formed the legal basis for official establishment of drug regulation in the history of Ethiopia. The Pharmacy and Laboratory Department under the then Ministry of Health was responsible for medicines regulation until June 1999 when a new regulation called the “*Drug Administration and Control Proclamation No. 176/1999*” was promulgated on 29 June 1999. Following this proclamation, the regulatory component of the Pharmacy Department was transformed to an independent Drug Administration and Control Authority (DACA) of Ethiopia in September 2001 (34).

Following the collapse of the Dergue regime in Ethiopia, the pharmaceutical sector has experienced significant expansion and progress, resulting in a majority of pharmaceuticals and medical supplies being supplied by both public and private entities. Presently, there are 32 plants, varying in size, engaged in the production of pharmaceuticals and associated products, with only 12 focusing on generic finished pharmaceutical dosage forms. The rest are dedicated to the small-scale manufacturing of medical devices, supplies, laboratory reagents, cosmetics, and disinfectants (35). As per the EFMHACA website, Ethiopia currently has 133 importers, 272 wholesalers, 377 pharmacies, 1,699 drug shops, and 1,392 rural drug vendors. An effective pharmaceutical legislation establishes regulatory authorities to enforce regulations. EFMHACA in Ethiopia is authorized by the Council of Ministers Legislation 189/2010 to serve as the regulatory authority for medicines. “Currently, the Ethiopian Food, Medicine, and Healthcare Administration and Control Authority (EFMHACA) have been restructured to the Ethiopian Food and Drug Authority (EFDA). The organizational structure of this regulatory authority, shown in Supplementary File S1, demonstrates how its various components coordinate to ensure that pharmaceutical services adhere to regulatory standards effectively.

### Regulatory requirements for medicine retail outlets in Ethiopia

In Ethiopia, there is a promising activity implemented by Ethiopian Food and Drug Authority (EFDA) with regard to premise licensing of drug outlets through risk based analysis (36). However, when compared to other African countries, the country’s pharmacy practice is not as developed (37). Despite some regulatory compliance issues, the Ghana Health Professionals Regulatory Bodies Act of 857, 2013 ensures inspections of pharmacies and OTC medicine facilities, conducting routine checks and offering technical support to practitioners (38). In contrast, Ethiopia’s pharmaceutical system requires further efforts from the Ethiopian Food and Drug Authority (EFDA) to enhance regulatory performance, though it is making promising progress (36, 39, 40). In Ethiopia, the main law governing pharmacy practice is the Health Professions Regulatory Bodies Act, Act 857, 2013, with additional regulations applicable to the pharmaceutical sector. According to this law, the private healthcare providers and medicine retail outlets must adhere to minimum regulatory standards (40) (Table 1).

**Table 1:** Regulatory requirements for a medicine retail outlet in Ethiopia (39).

Standards	Some specific requirements
Premises and facilities	The premises on which a dispensing service is provided would reflect the quality of service and inspire confidence on patients in the nature of pharmaceutical service delivered
	The walls, floors, windows, ceiling, and all other parts of the premises should be as per the requirement set by the regulatory body
	All parts of the premises are recommended to be maintained in an orderly and tidy condition
	The dispensing environment should possess appropriate temperature, sufficient lighting, humidity control, cold storage facilities, adequate shelving to ensure the integrity of the stored drugs, a dispensing table, and aids.
Storage conditions	Normal, cold, and special storage conditions should be used
	Refrigerators that open on the top are more efficient than vertical ones, because hot air rises while cold air falls
	Store products that are sensitive to freezing or very low temperatures on the upper shelves.
	If there is enough space, place a few plastic bottles of water in the refrigerator. This will help maintain the temperature for a longer period of time if the power is cut off
Dispenser	A person who is authorized to dispense drugs and medical supplies to recipients
	Depending on the level of dispensaries, pharmacy professionals of varying level of qualification may be licensed for dispensing practices.
	All licensed private pharmacies, drug shops and rural drug vendor shops are required to work under the technical leadership of registered pharmacists, druggists and pharmacy technicians, respectively, as per the new proclamation
	The drug outlets within public health institutions are to be managed by appropriately qualified staff: a pharmacist, a druggist, or pharmacy technician-depending on the level of the outlet.
Hygiene and sanitation	The Physical surroundings must be maintained as free of dust and dirt as possible.
	Maintaining a clean environment requires a regular routine of cleaning shelves and a daily cleaning of floors and working surfaces
	Dispensing equipment used for measuring liquids or counting tablets or capsules should be kept clean at all times

## Materials and methods

### Literature search strategies

The data for this study were extracted from PubMed, Web of Science databases, and Regulatory Website like Ethiopian Food and Drug Authority using a combination of keywords, including “regulatory compliance” AND “pharmacy outlets” OR “drug” OR “medicine” AND “Ethiopia,” “poor drug quality” OR “substandard” OR “falsified” AND “drugs” OR “pharmaceutical preparations,” OR “pharmaceutical” AND “preparations” OR “pharmaceutical preparations” OR “drugs” AND “regulatory compliance OR “non-Compliance.”

The published data were reviewed to collect information regarding regulatory compliance. The review aimed to identify gaps in compliance among medicine retail outlets and develop recommendations for improving future regulations. Review questions were designed to address areas inadequately explored in previous research, and potential opportunities for future studies were identified to expand and deepen the existing knowledge base on regulatory compliance in medicine retail outlets.

The literature review aimed to address three key review questions to evaluate the regulatory compliance of medicine retail outlets and the quality of pharmaceutical products in the Ethiopian pharmaceutical market. These review questions were: (1) What is the current status of regulatory compliance of medicine retail outlets with the Ethiopian Food and Drug Authority (EFDA) regulatory system? (2) What is the quality status of pharmaceutical products found in medicine retail outlets? (3) What future policy strategies are being implemented in medicine retail outlets to address and combat the presence of non-compliance with regulatory instruments?

To ensure information validity, the review incorporated literature published in indexed, peer-reviewed journals, thereby guaranteeing scientifically reliable recommendations. Studies from non-indexed journals were excluded. For the purpose of comparison and identifying gaps in the enforcement of regulatory standards in medicine retail outlets, data from both developed and developing countries, including Ethiopia, were included (8–11, 13, 14, 18, 31, 41–43). The regulatory enforcement experiences from other countries were analyzed to identify regulatory enforcement differences, providing insights into areas where Ethiopia’s regulatory framework could benefit for improvement.

### Operational definitions

#### Medicine retail outlets

These are private, government, or non-government-owned facilities operated by a registered and licensed pharmacist, druggist, or Level IV pharmacy technician.

#### Good storage practice

The adherence of medicine retail outlets to the storage standards specified by the Ethiopian Food and Drug Authority (EFDA).

#### Noncompliant

Medicine retail outlets that fail to meet the regulatory standards set by the Ethiopian Food and Drug Authority.

#### Compliant

Medicine retail outlets that demonstrate effective implementation of the pharmaceutical regulatory guidelines, thereby meeting the regulatory standards.

#### Poor quality medicines

Medicines that are substandard, falsified, or degraded failing to meet the official quality standards set for them.

### Literature search results

The literature review on medicine retail outlets and their compliance with the Ethiopian Food and Drug Authority (EFDA) regulatory standards indicates significant challenges in the pharmaceutical market in Ethiopia (39).

### Intricate supply chain systems and regulatory deficiencies

Complex supply chains and inadequate regulatory measures contribute to non-compliant pharmacies and the distribution of substandard medications (44). Lack of regulatory enforcement leads to the production and distribution of low-quality drugs, which adversely impact public health and strain the health budget within Ethiopia, contributing to the existence of non-compliant pharmacies and the distribution of substandard medications in the pharmaceutical markets (14). Health facilities that do not comply with the regulatory system not only contribute to the production of low-quality drugs but also impact the health budget when they fail to adhere to proper dispensing practices.

### Drug expiration and monetary loss in health facilities

Diriba et al. reported a 5% expiration rate in the Western cluster of pharmaceutical supply chain organizations during 2012–2013, with expired drugs valued at 20 million Ethiopian Birr (ETB) (44). Furthermore, in the fiscal years 2019/2020 and 2020/2021, Getahun et al. discovered that the medicine waste rate averaged at 4.87% in the public hospitals of Jimma Zone, amounting to a total value of 32,453.3 US$ (45). Moreover, an approximate sum of 2711.44 US$ was expended by the facility for the disposal of expired medicines in the same hospitals (45).

The report from Ethiopia revealed the significant issue of medicine wastage in Dire-Dawa public health facilities between 2010 and 2012 EFY. The average wastage rate was 3.07%, resulting in a financial loss of 4,048,594.0 ETB. The most wasted class of medications was anti-infective drugs, which accounted for 2,360,330 ETB (58.3%) of the total wastage. Furthermore, tablets represented a large proportion of the wasted medications, amounting to 2,615,391 ETB (64.6%) (46). This wastage underlines significant inefficiencies in the management of pharmaceuticals within health facilities. One critical factor contributing to this issue is the lack of regulatory standards for recycling surplus drugs. Without clear guidelines or systems for redistributing unused medicines, excess stocks often go to waste, leading to financial losses and reduced access to essential medications for patients (47).

### Adherence to controlled prescription regulations, and regulatory compliance

A thorough analysis of published literature was conducted to investigate whether drug stores are fit with the Ethiopian Food and Drugs Regulation System. This review focused on medicine retail outlets (MROs) and their interactions with the regulatory system. A report from Gondar and Bahir Dar towns in the Amhara region of Ethiopia indicates a non-compliance issue of medicine retail outlets. The findings revealed that the overall adherence to Ethiopia’s controlled prescription regulation was inadequate, with only 23.9% (SD = 18.3%) compliance observed across all 107 medicine retail outlets surveyed for five different drugs. Particularly concerning was the remarkably low compliance rates for strong narcotics, with pethidine at 3.3% and morphine at 8% (42).

According to the Ethiopian Narcotic Drug Control Regulation, it is mandatory for every private wholesaler or dispenser to report the stock status every 3 months. Despite this, there are instances where pharmacy professionals acquire narcotics drugs without adhering to the regulatory framework. A study revealed that two drug outlets had previously been sanctioned by the control authority for violating the rules of drug regulation. Additionally, 5% of professionals working in private pharmacies are aware of colleagues who obtain narcotics drugs outside of the regulated supply chain, although they claim to be free of such misconduct (42).

According to a study conducted in Jimma, it was found that approximately 45% of drug retail establishments did not meet the required regulatory standards (14). The study also identified several factors that influenced the level of regulatory compliance, including the income of the retail outlet, the experience of the dispenser, and the training received on good storage practices. These factors showed a significant association with regulatory compliance status, as indicated by a *p*-value of less than 0.05. The findings indicate that the private drug retail outlets in Jimma town do not meet the required standards of regulatory compliance (14). Additionally, study conducted in the western Wollega zone of Ethiopia, it was found that out of the 23 health facilities that were assessed, 17 (73.91%) (including 4 (100%) hospitals) and 13 (68.42%) health centers met the desired storage conditions (48). The storage conditions in hospitals’ stores, which were equipped with furniture and equipment, were found to be satisfactory. However, a considerable number of health centers’ stores did not meet the desired storage conditions (48). The investigation suggests the Ethiopian Food and Drug Authority to conduct inspections of the healthcare facilities in accordance with the regulatory guidelines.

Non-compliance problems are prevalent not only in terms of drug outlets but also among healthcare professionals in Ethiopia. A study by Dejene et al. surveyed 554 health professionals, finding that 32.5% were unregistered, and 72.8% failed to renew their licenses. Additionally, ethical breaches were not addressed, and 97.8% never identified their own continuing professional development (CPD) (49). The study also revealed concerns about skilled staff shortages, budget constraints, and inadequate infrastructure, which are hindering regulatory enforcement.

### The potential impact of pharmaceutical regulatory system on the quality of medicine

According to the literature, the presence of substandard drugs is expected when the regulatory system of a country is weak (6, 14, 25). Specifically, the presence of weak pharmaceutical regulatory performance in Ethiopia has resulted in the proliferation of substandard medications within the pharmaceutical sector. For instance, evidence showed Ethiopia exhibited the largest proportion of research studies on substandard and falsified drugs [9 out of 15 (60%)], with Kenya coming in second [4 out of 15 (26.6%)], and Tanzania and Rwanda each contributing 1 out of 15 (6.7%) (25). This may be related to the presence of non-compliant drug outlets in the study area.

**Figure 1:**
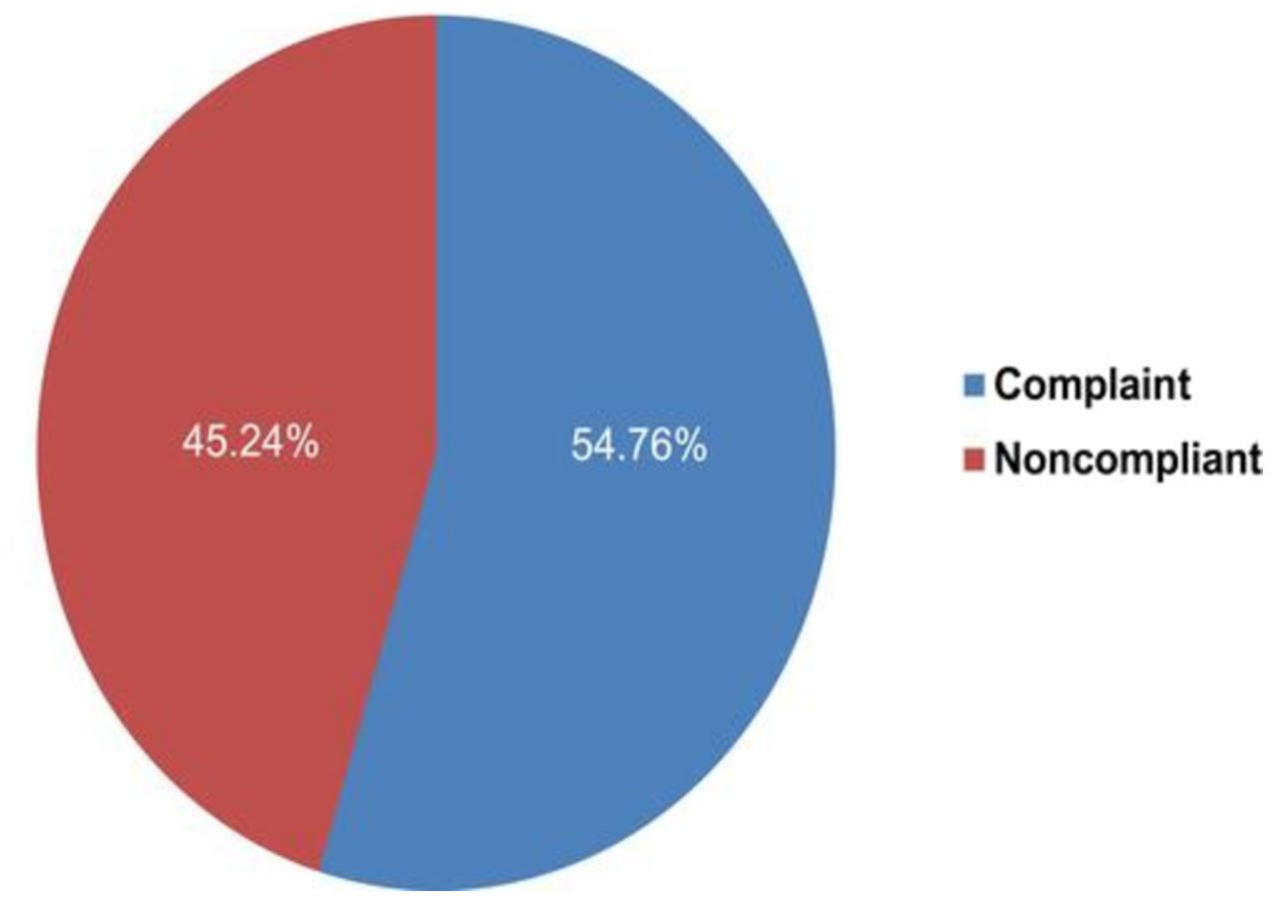
Status of regulatory compliance drug retail outlets in Jimma town pharmaceutical market.

The study performed by Aman et al. mentioned that the regulatory compliance of private drug retail outlets in Jimma town is not satisfactory, and the assays of amoxicillin obtained from noncompliant retail outlets appear to be slightly degraded, which may potentially demonstrate the impact of non-compliance of the drug retail outlets on the quality of medicines (14). Furthermore, the findings revealed that the presence of non-compliant outlets may result in degradation of drugs, which affects the general public. The report indicates only 54.76% were compliant with regulatory standards (14) (Figure 1).

A study in Pakistan inspected 1,003 drug stores, finding only 4.1% compliant with regulatory requirements (43). The majority sold general items, with only 12% having qualified staff. Additionally, about 47.4% displayed drug sales licenses, while 33.4% had expired licenses. Only 11.4% sold vaccines without proper refrigeration, and only 40.2% were adequately protected from sunlight. Only 5.4% had air conditioning installed. It is evident that there is an urgent requirement to enhance storage procedures in pharmacies by adhering to the regulatory guidelines and laws set forth by the drug regulatory authority of the respective nation in order to avoid delivering substandard pharmaceutical services.

In Bhutan, a study conducted in the South Asian region revealed that approximately 32.99% of community pharmacies lacked proper protocols for the storage and distribution of medicines. This highlights significant gaps in adherence to pharmaceutical guidelines, potentially impacting the quality, safety, and efficacy of medications distributed within the region (50). However, it was observed that all community pharmacies possessed the necessary technical authorization from the Drug Regulatory Authority. As the study found that the occurrence of “substandard and falsified medical products” obtained from community pharmacies ranged from 1 to 5% (51). Among these, erectile dysfunction medications were at the highest risk of being falsified. Any variations in packaging could potentially lead to suspicions. Out of the total, five (4.9%) cases of SFs were detected (*p* < 0.001 one-sample binomial test) (51). Among these, three (2.9%) were reported to the medicine agency. None of the cases were associated with any public health initiatives.

### Availability of dispensing and storage facilities

The distribution and storage of drugs are a significant aspect of healthcare, and it requires management support in the form of planning, funding, information management, and sufficient human resources (52). The storage space needs to be well-guarded, physically robust, and enough for secure handling and storage (53). For precise and secure operation, storage rooms require adequate lighting; secured, clean, dry, and temperature-controlled storage facilities and buildings must also be built or modified to ensure the best storage conditions (54). As per Ethiopian Food and Drug Regulations, drug outlets’ dispensing and storage facilities should be as per regulatory standards for delivering safe, effective, and qualified pharmaceutical services (35).

The study conducted in Amara regional state indicated that, the majority (53.4%) of pharmacy facilities lacked air conditioning and non-compliant to regulatory standards (54). Additionally, 1.9% of participants did not employ a strategy to arrange their medications. It is evident that pharmacy establishments require comprehensive regulatory oversight to ensure the secure provision of high-quality pharmaceutical services. A study on the compliance level of community pharmacies in Bhutan revealed that over 80% were found to be storing and dispensing medicines correctly. However, there are still various areas that require attention in order to achieve full compliance (50). Thus, regulatory compliance poses a universal challenge in the medicine retail outlet environment. Consequently, a collaborative effort between regulatory bodies, researchers, and pharmacy professionals should be initiated to combat non-compliance issue of MRO.

In the absence of a robust regulatory authority, issues such as incomplete prescriptions (54.34%) and illegible prescriptions (30.43%) were identified in community drug retail outlets, as reported by Demissie et al. in a study conducted in southwest Ethiopia, which evaluated pharmaceutical service quality in drug dispensing settings (55). The study indicates, dispensing errors, which subsequently result in drug resistance, adverse effects, and substandard pharmaceutical services (55).

### Medicine retail outlets and regulatory perspectives: past and future requirements

The review indicates ongoing challenges and future needs for improving pharmaceutical retail practices and regulatory frameworks. It also compiles the current regulatory compliance status and outlines future regulatory directions for medicine retail outlets, which were discussed, based on existing literature and input from regulatory agencies.

#### Current issues in medicine retail outlets

Many medicine retail outlets fail to comply with existing regulatory systems, which adversely affect the quality and safety of pharmaceutical products available in the market. Common issues include the distribution of inadequate doses, procurement of drugs from illegal sources, improper handling of medicines beyond the expertise of staff, and the provision of unauthorized medical services (9, 10, 14, 16, 18, 42, 48). A report from Ethiopia revealed that approximately 27.8% of drug dispensers lack professional qualifications, holding neither college diplomas nor university degrees. Investigations showed that dispensing practices at medicine retail outlets are largely non-standard. Key issues include the involvement of non-pharmacy professionals in dispensing, very short dispensing times, inadequate client knowledge, poor medication labeling, a high rate of dispensing errors, and the unavailability of essential dispensing aids (56). The study indicates the non-compliance of many medicine retail outlets with regulatory standards and emphasizes the need for strengthened regulatory efforts to ensure future improvements.

Additionally, the study noted that antimicrobial drugs retailed in private and government pharmacies in Ethiopia particularly in Adama town generally had long expiry dates, exceeding 6 months. For instance, 30 (78.9%) azithromycin and 38 (90.5%) ceftriaxone samples were found to have a long shelf life (57). This finding underlines the need for the Ethiopian Food and Drug Authority to conduct regular inspections of medicine retail outlets to monitor and ensure compliance with shelf-life requirements.

Study in Ethiopia has revealed that a significant proportion of retailers are involved in practices such as distributing drugs in inadequate doses (94%), handling drugs beyond their expertise (68%), and providing unauthorized medical services (63%). These findings emphasize the need for improved adherence to regulations and better practices in drug retail outlets (52). The inappropriate use of drugs in the private retail stores in the area is evident, as illustrated by the outcomes of this study (52).

#### Historical context pertaining to Ethiopia health settings

A study carried out in Southern Nations, nationalities, and people of Ethiopia revealed some concerning findings. The study discovered that significant proportions (94%) of the retailers were selling drugs below the necessary dosage (58). They justified this by claiming that customers were unable to afford the full dose. Additionally, 68% of the establishments were handling drugs that were beyond their level of expertise, including narcotics, hormones, and third-generation antibiotics. Furthermore, 63% of these establishments were providing unauthorized medical services on their premises. This indicates that the premises found in the facility failed to meet the required standards outlined in the regulations (58).

What will be needed in the future to enhance drug retail outlets? Literature suggest that the development and enforcement of new drug laws and regulations, along with educational interventions, will aid in encouraging rational practices (8). Additionally, rules and ethical standards governing the prescription and distribution of medications in both public and private settings are essential for informing potential regulatory actions (55, 57). Also, educational approaches may appear impactful in pharmacy outlet establishments; the dissemination of unbiased drug information and the organization of regular workshops can lead to incremental improvements in the practices of drug prescription and distribution among pharmacists (59).

A review conducted by Riley P., Callahan S., and Dalious M. in 2017 sheds light on the regulation of drug shops and pharmacies relevant to family planning, which contains a scan of 32 developing countries (60). Private pharmacies and drugs stores are an important source of modern family planning services and products in low-and middle-income nations. Donors and governments in many countries are looking into methods to enhance the role of drug retail stores, which must begin with an awareness of the legal and regulatory framework (61). According to World Health Organization (WHO) principles for effective pharmacy practice, governments should create a legal framework that specifies the scope of activity, determines who can practice, establishes competency criteria, and assigns resources to assure compliance (61). The Existed evidence indicated that laws for drug shops and pharmacies were not uniform, with significant variances in the level of detail provided in the guideline (62).

#### Scrutinizing regulatory gaps for future roadmap

Developing a future regulatory roadmap with targeted mitigation activities to ensure proper storage conditions and improve the quality of pharmaceutical services in health facilities is essential (63). The evidence highlights that providing compliance-related regulatory information for medicine retail outlets is vital, with significant implications for the pharmaceutical landscape (39). By addressing existing gaps in regulatory adherence and promoting best practices, the need for strategic interventions to enhance pharmaceutical service delivery in medicine retail outlets as per EFDA regulatory guideline (6).

Despite regulatory inspections by the country’s health authority, gaps in enforcement remain. For instance, a report from the Western Zone of the Tigray region revealed that about one-third of patients’ charts did not comply with Ethiopia’s standard treatment guidelines for general hospitals (64). By 2025, the Ethiopian government and health authorities aim to produce 1,500 graduates in industrial pharmacy and regulatory sciences (65). This strategy is expected to transform Ethiopia’s pharmaceutical sector, driving growth and development as a key economic engine.

#### Strengthening regulatory enforcement

There is a critical need to enhance the enforcement of regulatory standards across all levels of the pharmaceutical supply chain to ensure the distribution of high-quality medications (6, 8). Strengthening the regulatory framework is essential to ensure consistent enforcement of standards across all pharmaceutical outlets (57). This would involve regular inspections, monitoring, and penalties for non-compliance, which could lead to improved quality control and safer pharmaceutical products in the pharmaceutical market (24).

#### Combating drug expiration and pharmaceutical wastage

Implementing better inventory management practices and more rigorous monitoring can help reduce the rate of expired and wasted drugs, thereby saving costs and improving drug availability (66, 67). This approach not only saves costs but also improves drug availability, ensuring that medications are accessible to those who need them.

#### Improving compliance with prescription regulations

Increased oversight and stricter enforcement of controlled prescription regulations, especially for narcotics, are vital for restriction of non-compliance in private pharmacies (42). Implementing this strategy requires commitment and coordination among regulatory bodies, pharmacies, healthcare providers, and law enforcement agencies. Through a comprehensive and collaborative approach, it is possible to achieve a high level of compliance with controlled prescription regulations and safeguard public health (68).

#### Enhancing training and education

Providing continuous training on regulatory standards, good storage practices, and rational drug use for pharmacy staff is critical for enhancing compliance and the quality of pharmaceutical products (69). This endeavor ensures that pharmacy staffs are up-to-date with the latest regulations and best practices, thereby reducing the risk of dispensing substandard or counterfeit medications. It also promotes better handling and storage of pharmaceuticals, which can prevent degradation and ensure that patients receive effective treatments (69).

Additionally, educating pharmacy staff on rational drug use helps in minimizing medication errors and promoting the appropriate use of medicines, ultimately leading to improved patient outcomes and safety. Evidence indicate, an implementing such training programs can create a more knowledgeable and competent workforce, which is essential for maintaining high standards in pharmaceutical services (70). Furthermore, a study conducted in pharmacies, drug stores, and drug vendors in the South Gondar Zone of the Amhara Regional State revealed that dispensing medications without a prescription was a common practice in community drug retail outlets. The findings also indicated that dispensers often failed to conduct detailed assessments of patients’ signs and symptoms to accurately understand diarrheal cases (71). These issues highlight the need for targeted training for pharmacy professionals and the implementation of stricter regulatory controls.

#### Improving pharmaceutical storage practices

Ensuring that all health facilities, particularly health centers, meet the required storage conditions is indeed essential for maintaining the efficacy and safety of stored medications (12)`. Proper storage conditions, including temperature control, humidity management, and appropriate shelving, play a critical role in preserving the stability and potency of pharmaceuticals (72, 73). When medications are stored correctly, it helps to prevent degradation, contamination, and the potential for reduced effectiveness, which can compromise patient safety and treatment outcomes (48). By prioritizing potential strategies, health facilities can significantly improve the quality of pharmaceutical services and ensure that patients receive safe and effective medications (39).

#### Regulating healthcare professionals and continuing professional development

Enforcing registration and license renewal among healthcare professionals, addressing ethical breaches, and promoting Continuing Professional Development (CPD) are indeed crucial steps for maintaining professional standards and improving patient care (74). It is important to enforce the registration and renewal of licenses for healthcare professionals by ensuring that they are registered with the relevant regulatory bodies, setting up a system for timely renewal with reminders and deadlines, conducting regular verification checks to confirm that all practicing professionals have valid and up-to-date licenses, creating an automated system for registration and renewal with notifications for upcoming expirations, carrying out regular audits to ensure compliance with registration and licensing requirements, and implementing penalties for non-compliance, such as fines or suspension of practice (75). Implementing this activities and strategies, healthcare systems can maintain high professional standards, foster ethical practices, and ensure continuous professional development, ultimately leading to improved patient care and outcomes.

## Conclusion and future direction

The review indicates significant gaps in the compliance of medicine retail outlets in Ethiopia with the regulatory standards set by the Ethiopian Food and Drug Authority (EFDA). In low-and middle-income countries (LMICs), medicine retail outlets serve as key initial points of healthcare, yet their lack of adherence to regulations and absence of qualified staff compromise service quality and public health. One major consequence is the distribution of substandard drugs, which endangers the well-being of the population and diminishes trust in healthcare systems.

The review emphasizes the importance of implementing drug legislation and regulations alongside educational interventions to promote rational practices in medicine retailing. The implementation of regulatory standards for prescribing, dispensing medicines, storing, and following good transportation of medicine is critical for maintaining the quality of medicines and pharmaceutical services in health facilities. Additionally, the impartial dissemination of drug-related information and organizing periodic training workshops for pharmacy staff are recommended strategies to enhance prescription, dispensing and distribution practices. Overall, ensuring compliance with regulatory standards and improving the operational environment of medicine outlets are critical for safeguarding the public health, and safety. Addressing these issues through a combination of legal, educational, and ethical measures is vital for fostering a trustworthy and effective pharmaceutical service as per regulatory standards.

### The limitation and strength of the review

The review presents groundbreaking concepts on the adherence of medicine retail outlets to regulatory standards in Ethiopia. It also sheds light on the research void within medicine retail outlets, where extensive exchanges of drug information, which are relevant in disease environments and for the business sector in the pharmaceutical market, take place.

The review acknowledges a key limitation in its narrow focus on EFDA, which restricted the ability to assess the broader implementation of regulatory standards of other African regions. The review provides valuable insights derived from a cross-sectional analysis of medicine retail outlets’ compliance with regulatory standards. Additionally, this review primarily focused on a specific quality control tool applied to drug laboratory testing and evaluated the quality of drugs based on selected criteria. It compared non-compliant and compliant medicine retail outlets to gauge the effectiveness of these regulations in ensuring drug quality.

Overall, the limitation of the study’s scope, particularly in linking cross-sectional findings with quality control parameters from both compliant and non-compliant outlets, may have influenced the review’s conclusions. The review underlines the need for broader, more comprehensive research in the future that could provide a stronger and more extensive assessment of the overall effectiveness of regulatory standards and enforcement across medicine retail outlets.
