# Supplementary figures and images for: Situational analysis and future directions for medicine retail outlets: compliance with pharmaceutical regulatory standards in Ethiopia
Source: Front Med (Lausanne). 2025 Mar 12;12:1452875. doi: 10.3389/fmed.2025.1452875 (PMC11956760; doi:10.3389/fmed.2025.1452875)

# Ethiopian Food and Drug Authority - Organogram

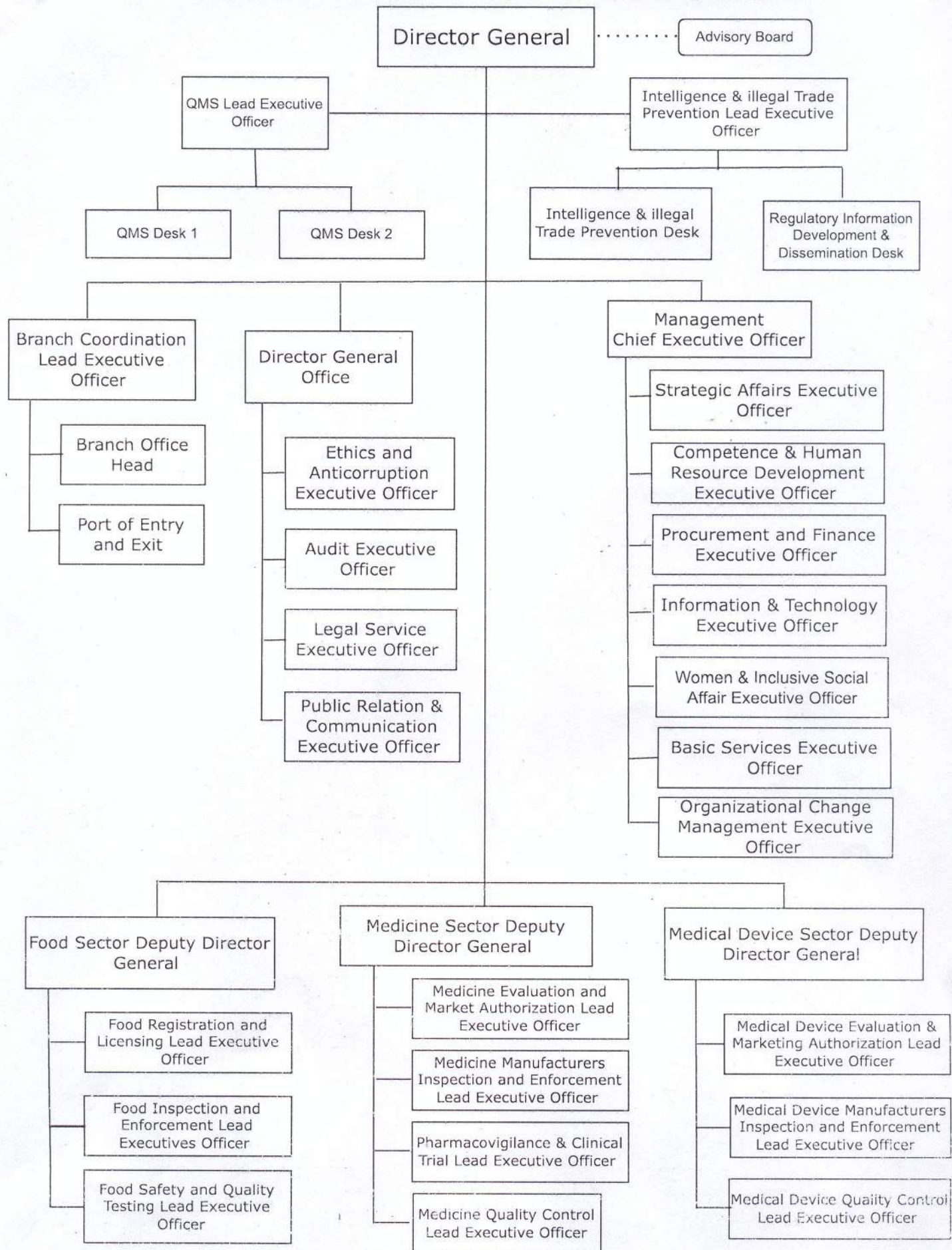

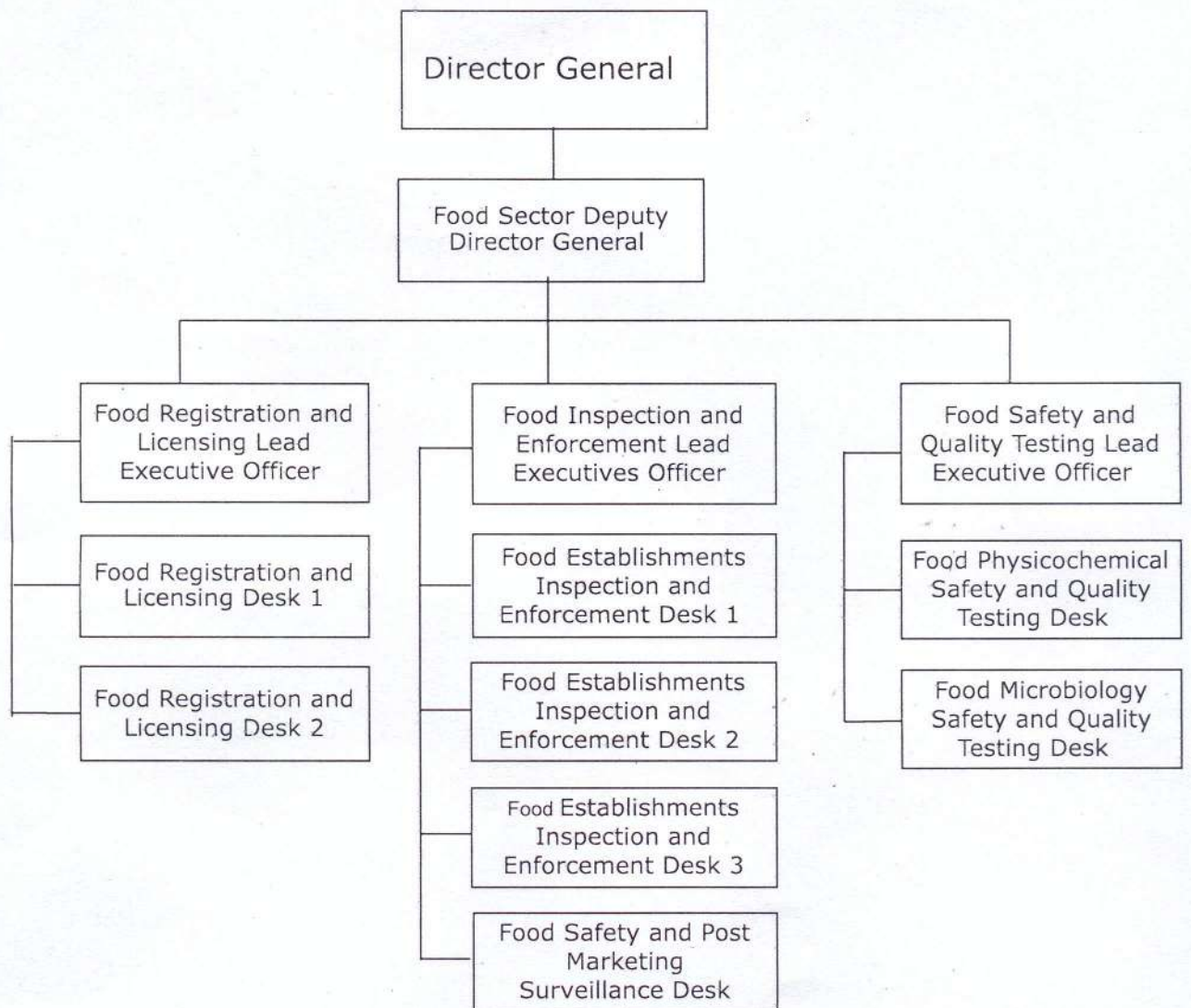

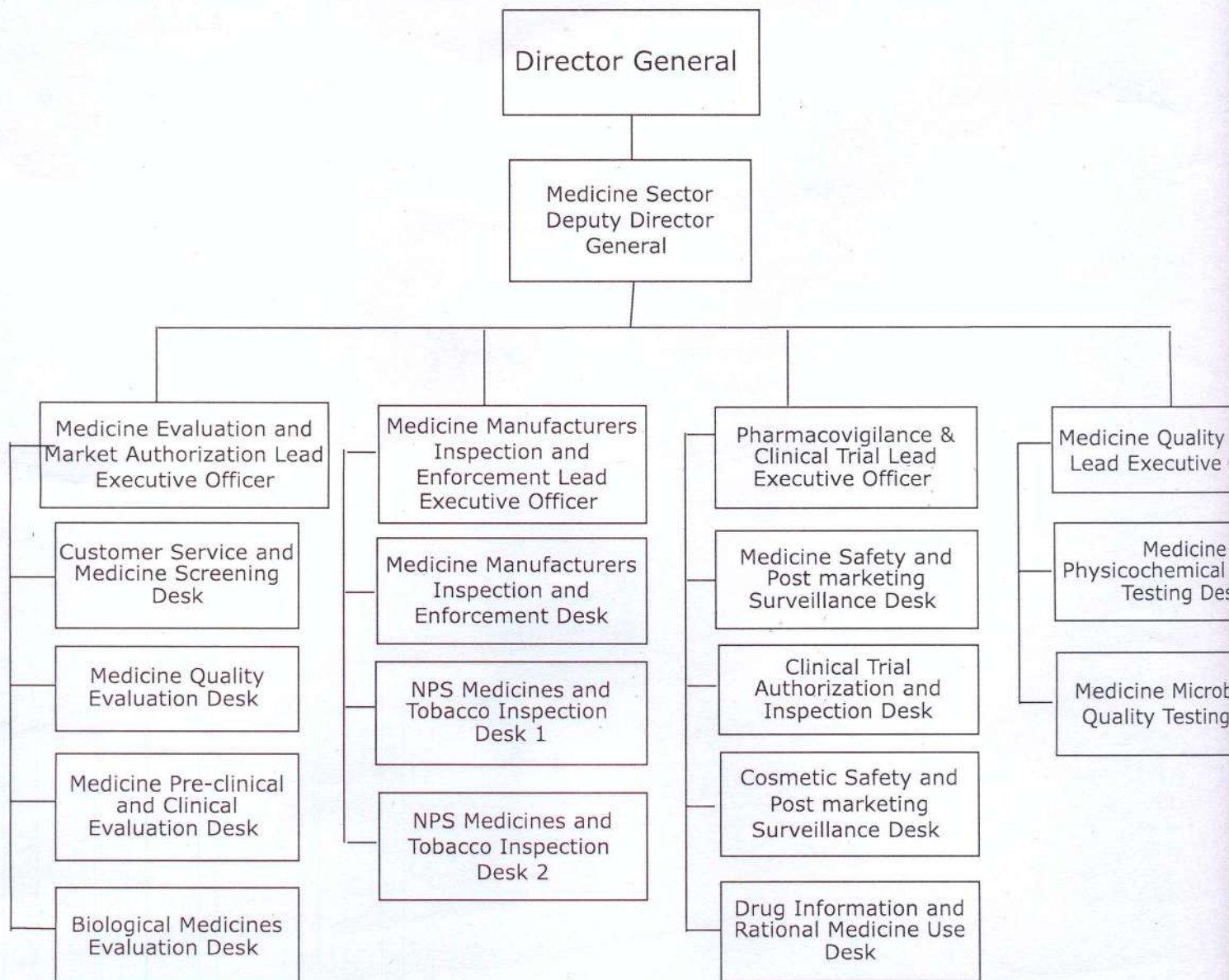

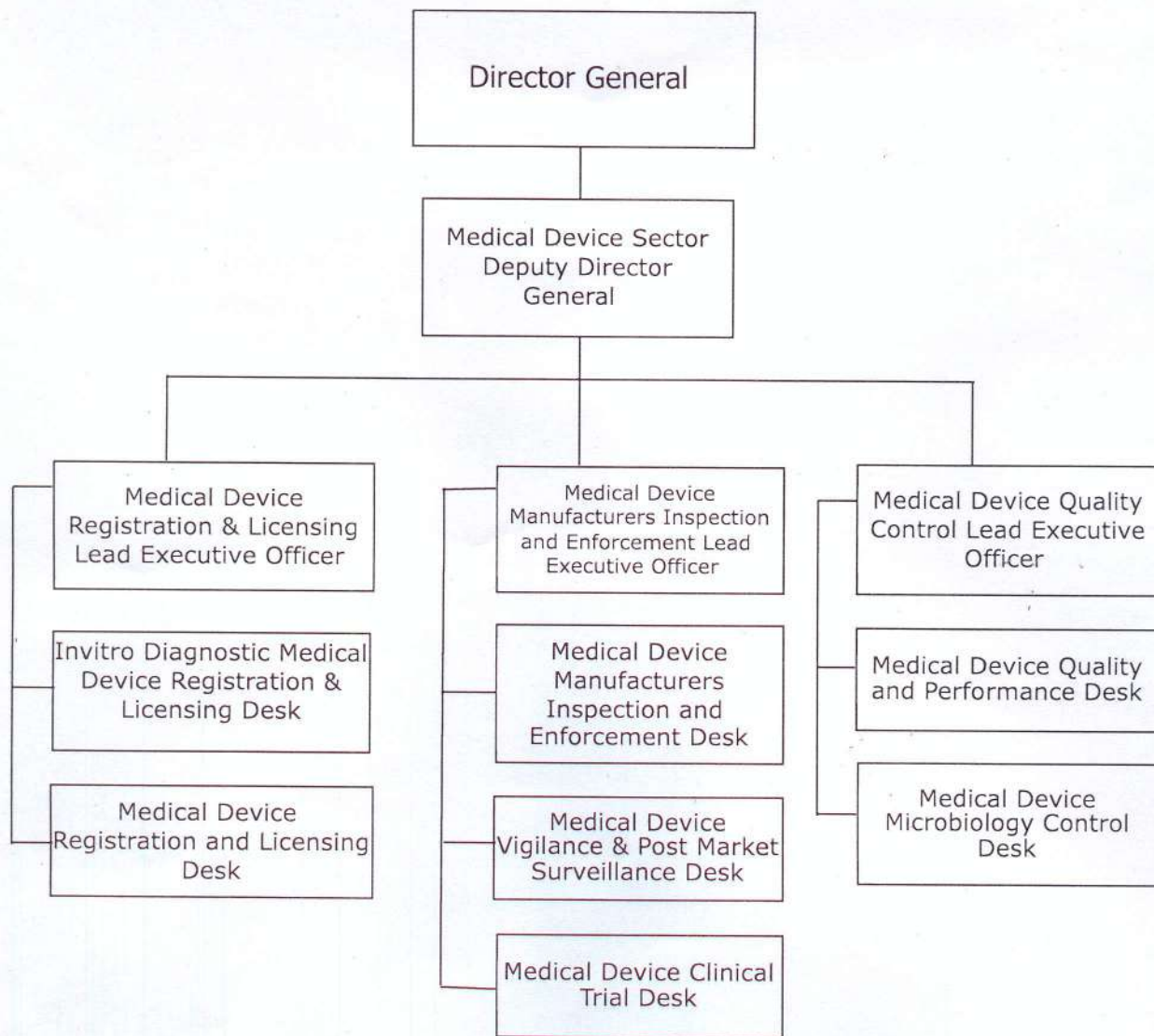

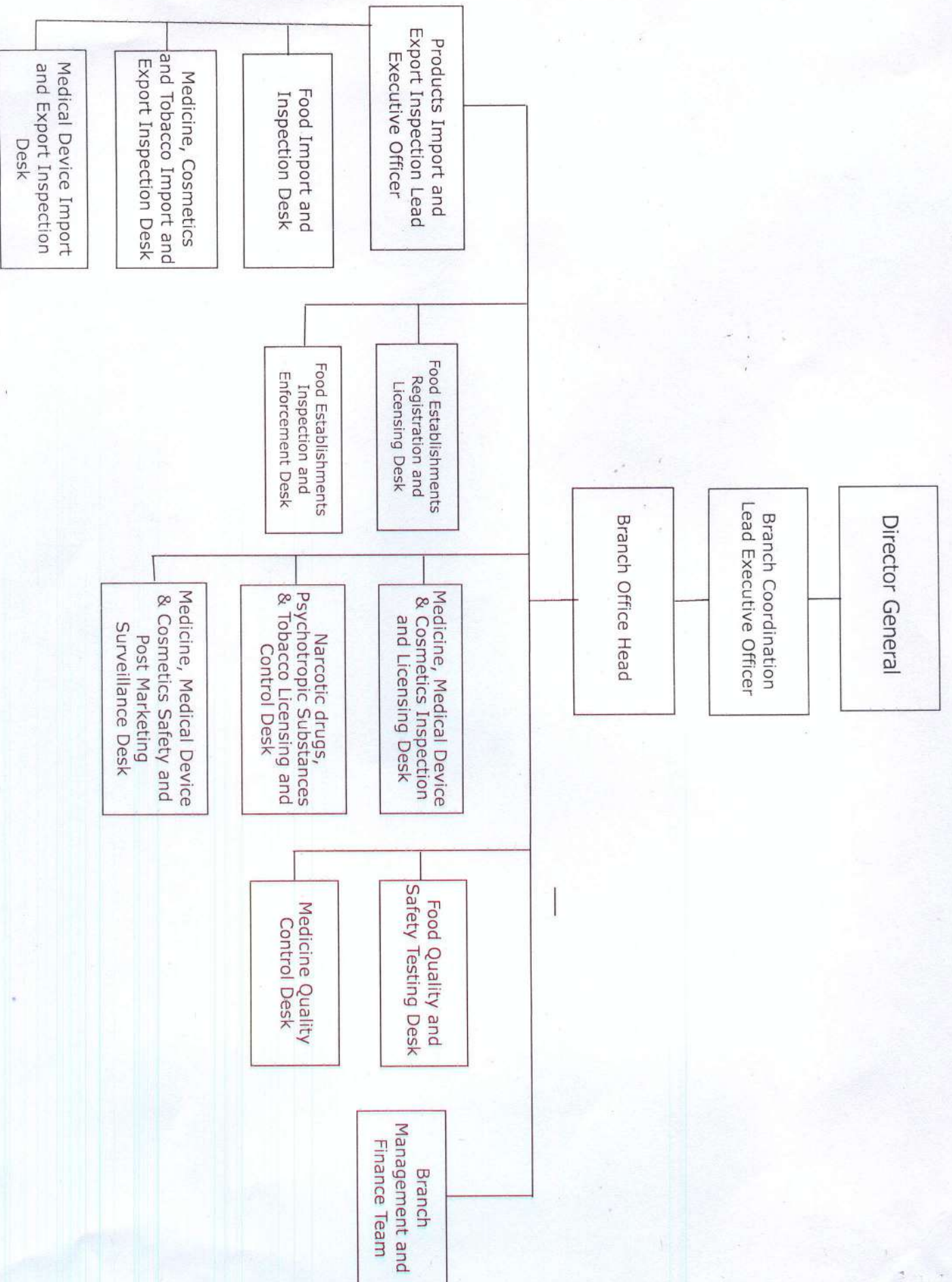

Supplement: Supplementary file 1 [file Data_Sheet_1.pdf]
